# Are Online Social Experiences Associated With General Interpersonal Problems? A Circumplex Assessment

**DOI:** 10.1002/jclp.70142

**Published:** 2026-04-03

**Authors:** Timothy W. Smith, Giovanni Marquez, Dakota A. Dolister, Bert N. Uchino, Kevin D. Jordan

**Affiliations:** ^1^ Department of Psychology and Health Psychology Program University of Utah Salt Lake City Utah USA; ^2^ Department of Psychology University of Evansville Evansville Indiana USA

**Keywords:** interpersonal circumplex, interpersonal problems, on‐line experience, social media addiction, social media use

## Abstract

**Objective:**

Social media use can promote social connection but also often includes negative experiences, raising questions about its associations with broader interpersonal functioning.

**Methods:**

This preregistered study used the interpersonal circumplex (IPC) to examine associations of self‐reported online social support and negativity, and excessive social media use with self‐reports of general interpersonal problems, compared to associations for offline support and social anxiety, in a representative sample of U.S. adult social media users (*n* = 1356; mean age = 44.9; 52% women; 61.9% non‐Hispanic White; median income $50k–75k).

**Results:**

Online negativity and excessive social media use were strongly associated with more severe general interpersonal problems; online support had a small positive association with greater problems. These associations were stronger for men than women, and weaker for older participants. In contrast to online support, off‐line social support was inversely associated with interpersonal problems, and the positive association for social anxiety was similar in magnitude to those for online negativity and excessive social media use.

**Conclusions:**

Findings suggest that online social experiences and excessive social media use are associated with broader interpersonal difficulties, and that the interpersonal perspective may be useful in understanding psychosocial issues emerging in the digital age.

Over 70% of adults in the United States use social media on a daily basis (Kemp 2023; Pew Research Center 2021), and 3 billion people worldwide use Facebook (Dixon 2023). This extensive online activity provides many opportunities for social connection, which can promote emotional adjustment and physical health (Office of the Surgeon General 2023). Generally, access to and use of the internet is associated with greater well‐being, including satisfaction with social relationships and experiences (Vuorre and Przybylski 2024). However, problematic social media use (e.g., excessive online activity) and other negative technology‐mediated social exposures (TMSE) can also undermine well‐being (Meier and Reinecke 2021).

To date, the extent to which adverse TMSE and excessive social media use are context‐specific issues or are instead related to broader patterns of interpersonal functioning is not well‐defined. In a novel application of the interpersonal perspective in personality and clinical psychology (Wright et al. 2023), the present study utilized concepts and methods from this general framework to examine associations of online social support, online social negativity, and excessive social media use with general interpersonal problems.

## 1 Online Support and Negativity, and Excessive Social Media Use

Social media use can be a source of positive experiences, including social connection (Janicke‐Bowles et al. 2023). For example, more frequent social media use and greater online social support are associated with greater reports of general social support (Kent de Grey et al. 2019; Liu et al. 2018; Uchino et al. 2024) and less loneliness (Zhang et al. 2021). However, online social experiences can also be a source of stress (Hall et al. 2021; Khetawat and Steele 2023). Negative TMSE include cyberbullying, anxiety over approval and missing out, actual social exclusion, and exposure to racism (Chan et al. 2021; Hall et al. 2021; Giumetti and Kowalski 2022; Jane 2015; Kent de Grey et al. 2019; Keum and Miller 2017; Li et al. 2022). Also, social media use can reach levels that include addictive features, such as excessive and disruptive concern about TMSE and difficulties in self‐regulation of its use (Marino et al. 2018a).

These patterns of problematic social media use and aversive TMSE are associated with broader aspects of adjustment, including dysfunctional personality traits, greater distress and loneliness, and lower well‐being and social support (Marino et al. 2018a, 2018b; Mozafar Saadati et al. 2021; Nunez and Radtke 2024; Shannon et al. 2022; Uchino et al. 2024). In studies directly examining correspondence between online experiences and parallel aspects of general psychosocial functioning, online social negativity is associated with reduced reports of general social support and increased reports of aversive social experiences beyond the online context (Kent de Grey et al. 2019; Uchino et al. 2024). However, the extent to which positive and negative on‐line experiences and problematic social media use are related to individuals' broader interpersonal experiences and difficulties is not well‐documented in research to date.

## 2 The Interpersonal Perspective

**Figure 1:**
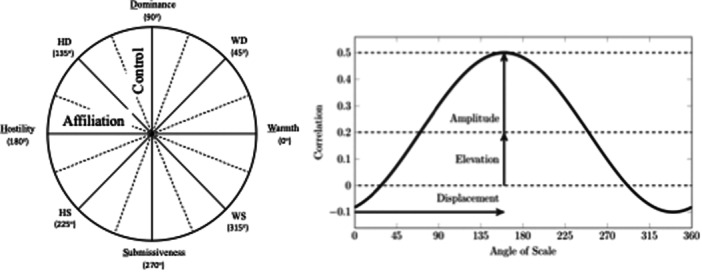
Interpersonal Circumplex (IPC; left panel) and Structural Summary Method (SSM: right panel). *Note:* In the left panel, the interpersonal circumplex (IPC) comprises two main dimensions, with octants reflecting blends of affiliation and control. HD, hostile dominance; HS, hostile submissiveness; WD, warm dominance; WS, warm submissiveness. IPC locations depicted in degrees, ranging counter‐clockwise from the warm pole. In the right panel, a hypothetical Structural Summary Method curve of correlations of a variable with IPC‐based reports of interpersonal problems, with the strongest positive association falling in the hostile‐dominant (HD) portion of the IPC.

Concepts and methods in the interpersonal tradition in personality and clinical psychology (for a review, see Wright et al. 2023) can provide a more definitive evaluation of this issue. The interpersonal circumplex (IPC; Figure 1, left panel) provides a framework and assessments that describe interpersonal experiences, behavior and other processes as blends of *affiliation* (i.e., warmth vs. coldness or hostility) and *control* (i.e., dominance vs. deference or submissiveness) (Fournier et al. 2011). Well‐validated measures assess interpersonal difficulties using this framework (Boudreaux et al. 2018; Soldz et al. 1995). These interpersonal problem inventories assess the extent to which individuals report problematic behavior or general difficulties reflecting various blends of affiliation and control depicted in Figure 1 (left panel). For example, in one widely‐used measure of interpersonal problems, excessive hostile‐dominance (e.g., vindictiveness) is reflected in the item, “I want to get revenge against people too much” (Soldz et al. 1995). In contrast, excessive warm‐submissiveness (i.e., exploitiveness) is reflected in the item, “I let other people take advantage of me too much” (Soldz et al. 1995).

The Structural Summary Method (SSM) of IPC‐based analysis (Zimmermann and Wright 2017) is an approach to evaluating interpersonal correlates (i.e., content or meaning) of a measure of interest, rather than a method of scoring. The related model posits that correlations of measures containing specific interpersonal thematic content with an IPC‐based inventory that includes octant scales form a sinusoidal pattern, also depicted in Figure 1 (right‐hand panel). The largest positive and negative correlations are at opposite IPC locations, correlations with adjacent octants are predicted to be smaller, and correlations that are orthogonal (i.e., 90° from the largest correlations) should be smallest. In SSM analyses, several meaningful parameters are derived from those correlations. *Elevation* represents the average correlation with IPC octants. For measures of interpersonal problems, elevation reflects the association with general interpersonal difficulties or distress (Tracey et al. 1996; Wendt et al. 2019) and is associated with many forms of maladjustment, with strong associations for internalizing disorders (Girard et al. 2017). *Amplitude* is the difference between elevation and the magnitude of association at the sinusoidal curve peak, and reflects the degree of specific interpersonal content. *Angular displacement* of this peak corresponds to the interpersonal theme of that specific content.

Thus, for SSM analyses of interpersonal problems, elevation would indicate the extent to which a social media measure (e.g., online negativity or excessive social media use) is related to overall interpersonal dysfunction or distress. If sinusoidal curve fit is adequate, amplitude values would indicate the extent to which the social media scale had a specific interpersonal theme or content beyond general interpersonal distress. Angular displacement would identify the theme of that specific interpersonal content, reflecting general interpersonal style as well as the content of the most pronounced interpersonal problems associated with the social media measure.

## 3 The Present Study

As an initial examination of the IPC framework for describing the extent to which measures of online experiences and problematic social media use are associated with general interpersonal difficulties, we examined their association with a well‐validated IPC‐based measure of interpersonal problems (Soldz et al. 1995). We predicted that both negative online experiences and excessive social media use would be associated with greater interpersonal difficulties (i.e., SSM elevation), as well as specific problems with excessive hostility and/or low warmth. In contrast, we predicted that higher reported online support would be associated with lower levels of interpersonal distress, as well as lower levels of specific problems with hostility.

Further, we compared IPC‐based correlates of online experience and social media use to other characteristics, specifically off‐line support (Cohen et al. 1985) and social anxiety (Brown et al. 1997). Social support is typically associated with higher warmth and lower hostility (Smith et al. 2010), less interpersonal distress, as well as lower levels of hostile interpersonal problems (Osman et al. 2014). Social anxiety is most commonly associated with a hostile‐submissive interpersonal style and problems (Alden and Phillips 1990), although there is heterogeneity in specific types of interpersonal problems socially‐anxious individuals report, such that it is also expected to be positively associated with overall interpersonal distress (e.g., Cain et al. 2010).

Evidence suggests that men are more susceptible to problematic internet use than are women (Su et al. 2019), and social media use may differ across adulthood, with important implications for the impact of TMSE among older adults (Cotten et al. 2022; Newman et al. 2021). However, associations of gender and age with online social negativity (e.g., cyberbullying) and other social media use have been inconsistent (Chan et al. 2021; Cotten et al. 2022), Hence, we also tested gender and age differences in online social experiences and problematic social media use, and in their associations with interpersonal problems.

## 4 Methods

### 4.1 Participants

As described elsewhere (Luchtefeld and Jordan 2022), a representative sample of adult participants (i.e., at least 18 years of age) who were self‐described social media users living in the United States was recruited. The final sample (*n* = 1356) was middle‐aged on average (*M* = 44.9, SD = 17.3, range = 18–91), and included slightly more self‐identified women than men (52% vs. 48%; two individuals identified as transgender, one as “other”). Supporting the adequacy of the sampling strategy, the race and ethnicity make‐up of the sample was comparable to the 2021 U.S. Census (61.9% non‐Hispanic white, 17.4% Hispanic, 12.3% non‐Hispanic black, 5.3% Asian, 3.2% American Indian/Alaskan Native). Regional composition of the sample was also consistent with the 2021 Census (21.3% Midwest, 18.0% Northeast, 37.3% South, 23.38% West). The median annual income range was $50,000 to $74,999.

### 4.2 Procedure

The study protocol was approved by the Institutional Review Board of Indiana State University (#1689180). A Qualtrics panel aggregator directed collection of an online sample representative of the US population. Potential participants were recruited via email, banner placements, social media, and e‐newsletters. They received online information about the study and questionnaires, and were informed that participation was voluntary and anonymous, and that participation could be terminated at any time. The Qualtrics survey included a double opt‐in procedure, ensuring voluntary participation. Participants' decisions to continue represented informed consent. Straight‐line responses and attention‐check questions identified participants for deletion, although information as to how many participants were eliminated in this way was not available. Qualtrics employs incentive scales in recruitment panels, with choices for participants among participation rewards (e.g., gift cards, partner products or services, charitable contributions).

### 4.3 Measures

The Short Form of the Inventory of Interpersonal Problems Circumplex Scales (IIP‐SC) (Soldz et al. 1995) was used to assess general interpersonal difficulties. The IIP‐SC includes eight 4‐item scales, corresponding to each of the IPC octants depicted in Figure 1, and asks respondents to indicate how distressing each problem has been using 5‐point Likert scales (1 = not at all; 5 = extremely). Example items include: “I try to control other people too much” (dominant or domineering); “It is hard for me to feel close to people” (hostile or cold); “It is hard for me to introduce myself to new people” (hostile submissive or socially avoidant); “It is hard for me to be firm when I need to be” (submissive or nonassertive); “I put other people's needs before my own too much” (warm or overly nurturant); and “I tell personal things to other people too much” (warm dominant or intrusive). Previous research has provided extensive evidence of expected structure (i.e., a total distress factor, and separate IPC affiliation and control dimensions), adequate reliability and construct validity (e.g., Hopwood et al. 2008; Wilson et al. 2013). In the present sample, internal consistencies for the octant scales ranged from *ω* = 0.78 to 0.89 (median *ω* = 0.83), and was *ω* = 0.98 for the total interpersonal problems score.

Online social support and aversive experiences were assessed via the Online Social Experiences Measure (OSEM; Kent de Grey et al. 2019), which inquired about experiences in online social networks (i.e., “Facebook, Instagram, Twitter, Snapchat, etc.”) during the prior month. Using a 5‐point, Likert‐type format (1 = very slightly; 5 = extremely), 20 items measured positive online social experiences and 20 items measured online negativity. The online support or positivity scale assesses the extent of exposure to encouragement, understanding, help, and appreciation (e.g., “Members of my online networks care about me as a person”). The online negativity scale assesses the extent of exposure to criticism, ostracism, or rejection (e.g., “Members of my online social networks act in unpleasant or angry ways toward me”). Prior research with this measure supports its two‐dimensional structure, reliability, and construct validity (Kent de Grey et al. 2019). For example, online positivity is more closely associated with general social support than with general negative interpersonal experiences, whereas the opposite pattern was found for the online negativity scale. Internal consistencies in the present sample were *ω* = 0.95 for positivity and *ω* = 0.97 for negativity.

The Bergen Social Media Addiction Scale (BSMAS) comprises six, 5‐point Likert items (1 = very rarely, 5 = very often). It is a modified form of the Bergen Facebook Addiction Scale worded to be applicable to use of a broader range of social media sites (i.e., “Facebook, Twitter, Instgram, and the like”) (Andreassen et al. 2016). An example item reads, “How often during the last year have you tried to cut down on the use of social media without success?” Prior research supports the structure and reliability of this single dimension measure (Andreassen et al. 2016). Internal consistency in the present sample was high (*ω* = 0.88).

A modified version of the Interpersonal Support Evaluation List (ISEL‐12) was used to measure participants' offline social support (i.e., “relationships you have *in person*”). Twelve 4‐point Likert‐type items (“definitely true” to “definitely false”) from the original scale (Cohen et al. 1985) were used, with the only modification being the instruction that participants should describe only offline social experiences. Prior evidence supports the structure, reliability, and construct validity of the total ISEL‐12 score as used here (Cohen et al. 1985). Items reflect appraisal (i.e., advice, guidance), belonging (empathy, acceptance), and tangible (help, assistance, aid) aspects of social support (e.g., “I don't often get invited to do things with others” (reverse scored)). The total scale had high internal consistency (*ω* = 0.85) in the present sample.

Social anxiety was assessed via short forms of the Social Interaction Anxiety Scale (SIAS‐6) and the Social Phobia Scale (SPS‐6) (Brown et al. 1997). The combined scale includes 12 5‐point Likert‐type items (“not at all characteristic or true of me” to “extremely characteristic or true of me*”*). An example reads, “I find it difficult mixing comfortably with the people I work with.” Internal consistency for the 12‐item total scale was high in the current sample (*ω* = 0.96). Although some evidence supports the distinction between social interaction anxiety and social phobia as assessed by the SIAS‐6 and SPS‐6 scales, their large correlation (*r* > 0.7) also supports the use of a total score to capture broadly defined, general social anxiety (Brown et al. 1997).

### 4.4 Statistical Analyses, Transparency, and Openness

In preliminary analyses using SPSS 28.0.1, internal consistencies were calculated as MacDonald's omega (Revelle and Condon 2019). Also, gender (women, men) by age (younger, middle‐aged, older) ANOVAs were conducted on OSEM‐Support, OSEM‐Negativity, BSMAS, ISEL‐12, and the combined SIAS‐SPS scores. Age groups were trichotomized as 18 to 34, 35 to 56, and 57 and older, to form similar group sizes. Univariate correlations of these measures with IIP‐SC octant and total scores tested simple associations of on‐line and general psychosocial functioning measures with the individual problems in the IPC framework. For analyses involving participant gender, the three self‐identified transgender or “other” participants were excluded.

In the primary analyses, associations of the OSEM‐Positivity, OSEM‐Negativity, and BSMAS with the IIP‐SC were calculated and compared using the SSM package for R (Zimmermann and Wright 2017), with bootstrapped 95% CIs. As noted previously, the SSM is not a method of scoring IPC‐based measures. Rather, it generates conceptually meaningful parameters from the pattern of correlations of an external measure (e.g., excessive social media use) with IPC octant scores. SSM elevation values indicate the association of the candidate scale with the IIP‐SC general factor or general interpersonal distress (Tracey et al. 1996; Wendt et al. 2019). Elevation values of |0.15| or greater indicate a notable association with this IPC general factor (Zimmermann and Wright 2017). The fit of correlations of a given measure with the IIP‐SC octant scores (see Figure 1, left panel) with the predicted sinusoidal curve (Figure 1, right panel) is evaluated as *R* ^*2*^. If fit with the predicted curve is adequate (i.e., *R* ^*2*^ ≥ 0.70), amplitude and angular displacement can be interpreted. Amplitude values of 0.15 or greater indicate a notable degree of specific interpersonal content (Zimmermann and Wright 2017). The interpersonal theme associated with that specific content corresponds to the angular displacement in degrees, with the IPC warm pole representing 0/360 (see Figure 1). Associations with IPC affiliation and control dimensions provide additional indications of the interpersonal theme. Thus, SSM elevation for a candidate scale reflects its association with overall severity of interpersonal difficulties, whereas angular displacement and associations with the IPC dimensions of affiliation (i.e., warmth) and control (i.e., dominance) reflect the interpersonal style associated with the scale (Wendt et al. 2019; Wilson et al. 2013), although interpretations of style require an acceptable SSM model fit. The SSM procedure also generates tests of differences in SSM parameters, using 95% CIs in paired comparisons; differences in the IPC correlates between scales were tested in this manner.

Exploratory analyses determined whether IPC‐based correlates of online measures were similar for women and men, and across age groups. List‐wise deletion of cases with missing data resulted in a loss of less than 2% of participants for all analyses. Previous research (Zimmermann and Wright 2017) suggests that the present sample size provides accurate estimates of SSM parameters, and estimates of the significance of parameter differences between scales. These analyses were preregistered, and data and example SPSS and R code for analyses described above are also available < 10.17605/OSF. IO/46RYB>. Item wording and scoring for each measure are available in the cited references.

## 5 Results

### 5.1 Preliminary Analyses

Correlations among the predictor variables are reported elsewhere (Uchino et al. 2024). In 2 (gender) × 3 (age group) ANOVAs of the positive online social experiences scale (see Table 1), older participants reported less positive experiences than younger and middle‐aged groups, *F*(2, 1347) = 13.86, *p* < 0.001, *η*_p_^2^ = 0.02. Men reported slightly more positive experiences than women, *F*(1, 1347) = 5.04, *p* = 0.025, *η*_p_^2^ = 0.004, and in a small gender by age interaction, this gender difference was reversed in the older group, *F*(2, 1347) = 7.81, *p* < 0.001, *η*_p_^2^= 0.011.

**Table 1:** Means (and standard deviations) for gender and age groups, for online and general psychosocial functioning measures.

Outcome	Women			Men		
	Younger	Middle‐aged	Older	Younger	Middle‐aged	Older
Online positivity	63.5 (18.5)	62.5 (21.2)	61.1 (20.7)	67.1 (18.6)	69.7 (18.5)	57.6 (20.0)
Online negativity	45.2 (18.8)	40.0 (19.4)	27.0 (10.0)	48.6 (19.7)	53.2 (22.8)	30.2 (16.4)
Social media addiction	16.7(5.4)	14.8 (6.1)	10.2 (4.2)	17.5 (5.1)	17.8 (6.4)	10.6 (4.8)
Offline social support	34.6 (7.5)	36.6 (7.5)	37.7 (7.8)	35.9 (7.1)	34.7 (6.5)	37.0 (8.0)
Social anxiety	31.8 (12.7)	25.5 (11.8)	18.7 (8.5)	29.0 (13.3)	30.1 (15.0)	18.7 (9.2)

Older participants reported less negative online experiences than younger and middle‐aged participants, *F*(2, 1347) = 127.39, *p* < 0.001, *η*_p_^2^ = 0.16, and men reported more than women, *F*(1, 1347) = 41.31, *p* < 0.001, *η*_p_^2^ = 0.03. In a small gender by age interaction, this gender difference was largest in the middle‐aged group, *F*(2, 1347) = 10.65, *p* < 0.001, *η*_p_^2^= 0.016.

The ANOVA of social media addictive behavior indicated that older participants reported lower levels of addictive behavior than younger and middle‐aged participants, *F*(2, 1347) = 127.39, *p* < 0.001, *η*_p_^2^= 0.159. Also, men reported higher levels than did women, *F*(1, 1347) = 41.31, *p* < 0.001, *η*_p_^2^= 0.03. In a small gender by age interaction, this gender difference was largest among middle‐aged participants, *F*(2, 1347) = 10.65, *p* < 0.001, *η*_p_^2^= 0.016.

Older participants reported somewhat more off‐line social support than did middle‐aged and younger participants, *F*(2, 1347) = 8.83, *p* < 0.001, *η*_p_^2^ = 0.013. In a small gender by age interaction, this age difference was slightly larger for women, *F*(2, 1347) = 5.60, *p* = 0.004, *η*_p_^2^= 0.008. Older participants also reported lower levels of social anxiety than did younger and middle‐aged participants, *F*(2, 1346) = 99.57, *p* < 0.001, *η*_p_^2^ = 0.13. In a small age by gender interaction, younger women reported greater social anxiety than younger men, and this gender difference was reversed in the middle‐aged group, *F*(2, 1346) = 10.53, *p* < 0.001, *η*_p_^2^= 0.015.

Correlations of online experience measures, excessive social media use, offline support, and social anxiety with total interpersonal problems and individual octant problem scores are presented in Table 2. All three online experience measures were positively associated with interpersonal problems, especially negative online experiences and excessive social media use. Social anxiety was also strongly associated with interpersonal problems, whereas offline social support as inversely associated. These correlations are the basis of SSM analyses reported next.

**Table 2:** Univariate correlations of the IIP‐CS short form octant scales with online social experiences, social media addiction, social anxiety, and offline social support scales.

Interpersonal problem scales	Online support	Online negativity	Social media addiction	Offline support	Social anxiety
Dominance	0.15**	0.70**	0.56**	−0.40**	0.65**
Hostile dominance	0.07*	0.69**	0.51**	−0.48**	0.68**
Hostility	0.02	0.59**	0.45**	−0.51**	0.68**
Hostile submissiveness	−0.01	0.56**	0.41**	−0.54**	0.73**
Submissiveness	0.07*	0.55**	0.44**	−0.43**	0.64**
Warm submissiveness	0.15**	0.63**	0.52**	−0.40**	0.67**
Warmth	0.22**	0.50**	0.45**	−0.23**	0.50**
Warm dominance	0.26**	0.66**	0.56**	−0.27**	0.53**
Total problems	0.13**	0.71**	0.57**	−0.48**	0.74**

* *p* < 0.01

** *p* < 0.001.

### 5.2 Interpersonal Correlates of Online Experiences

**Figure 2:**
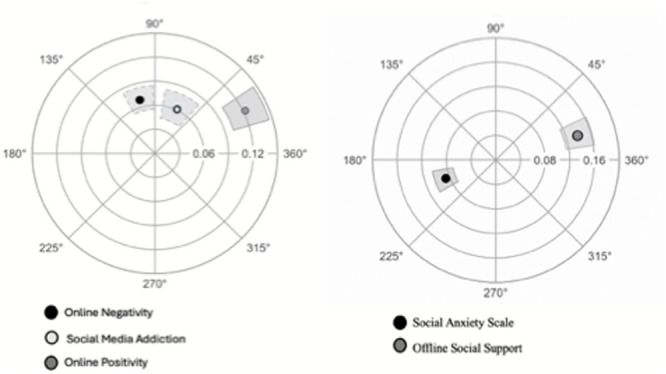
Interpersonal circumplex locations associated with on‐line experience (left panel) and off‐line measures (right panel). *Note:* Interpersonal themes (angular displacements) are in reference to the IPC pole of warmth (0/360). Strength of association with interpersonal content (amplitude) is reflected in distance from the origin (radius). Point estimates for these values are indicated, accompanied by 95% CIs (surrounding boxes) for amplitudes (radial CI) and angular displacements (angular CI). Dotted CI boxes indicate poor Structural Summary Method (SSM) model fit, which limits interpretation of amplitude and angular displacement values. Associations with overall severity of interpersonal problems (SSM elevation) are not depicted. See Table 3 for those results.

As presented in Table 3, online social support had a small, albeit significant positive elevation in the SSM analysis, reflecting its small but significant positive correlation with total interpersonal problems (see Table 2). The SSM model fit was excellent, and the amplitude suggests at least some specific interpersonal content. The angular displacement and associations with affiliation and control IPC dimensions suggest that the interpersonal style and major problem content associated with higher reports of online support is clearly warm and somewhat dominant (see Figure 2, left panel).

**Table 3:** Structural summary statistics with 95% CIs for associations of online social support, online social negativity, internet addiction, offline social support and social anxiety scales with self‐reported interpersonal problems.

Scale	Fit	Elevation	Amplitude	Displacement	Affiliation	Control
Online support	0.98	0.11 (0.06, 0.16)	0.12 (0.10, 0.14)	25.7 (16.9, 34.6)	0.11 (0.09, 0.13)	0.05 (0.03, 0.07)
Online negativity	0.53	0.61 (0.57, 0.64)	0.07 (0.06, 0.08)	104.6 (90.8, 117.4)	−0.02 (−0.03, −0.00)	0.07 (0.05, 0.08)
Social media addiction	0.64	0.49 (0.44, 0.53)	0.06 (0.04, 0.08)	63.9 (48.5, 81.6)	0.03 (0.01, 0.05)	0.06 (0.04, 0.07)
Offline support	0.91	−0.41 (−0.44, −0.37)	0.14 (0.12, 0.16)	17.2 (8.5, 25.6)	0.13 (0.11, 0.16)	0.04 (0.02, 0.06)
Social anxiety	0.70	0.63 (0.60, 0.66)	0.09 (0.07, 0.10)	199.6 (190.2, 209.5)	−0.08 (−0.10, −0.07)	−0.03 (−0.04, −0.01)

Online negativity had a large elevation value in the SSM analyses, reflecting its large correlation with total interpersonal problems. SSM model fit was poor, and amplitude was negligible, indicating that despite its strong association with general interpersonal distress or dysfunction, online negativity was not strongly associated with any specific interpersonal style.

Social media addiction severity also had a strong elevation in the SSM analyses, again consistent with its correlation with total IIP‐SC scores. Here, too a poor model fit and negligible amplitude value indicated no specific interpersonal theme beyond general interpersonal distress.

Follow‐up SSM analyses indicated that online social support had a substantial elevation, indicating an association with greater interpersonal problems, for men (0.21, CI: 0.14, 0.29) but not women (−0.01, CI: −0.07, 0.06). The elevation value for online negativity was also greater for men (0.68, CI: 0.63, 0.72) than women (0.52, CI: 0.47, 0.57), as was the elevation value for internet addiction for men (0.53, CI: 0.47, 0.59) versus women (0.42, CI: 0.36, 0.47), though unlike online support these associations were large for both men and women. Thus, the measures of online activity were somewhat more consistently and closely associated with overall interpersonal problems for men.

In follow‐up analyses of age group differences, the elevation value for online social support was larger in the middle‐aged group (0.24, CI: 0.16, 0.32) than among younger (0.03, CI: −0.07, 0.13) or older participants (−0.10, CI: −0.17, −0.02). That is, the association of online social support with greater general interpersonal difficulty was limited to the middle‐aged participants, and online support was associated with significantly lower levels of general interpersonal difficulties among older participants. Elevation values for online negativity were large for all three age groups, but higher in the younger (0.56, CI: 0.49, 0.62) and middle‐aged groups (0.67, CI: 0.61, 0.72) than the older group (0.35, CI: 0.25, 0.45). The elevation value for excessive internet use was greater in the middle‐aged group (0.56, CI: 0.48, 0.62) than in the younger (0.32, CI: 0.22, 0.42) and older (0.29, CI: 0.20, 0.37) groups. Thus, online activity had a generally weaker association with self‐reported general interpersonal difficulties for older participants, and a generally greater association with such difficulties among middle‐aged participants.

### 5.3 Interpersonal Correlates of Social Anxiety and Offline Social Support

Also presented in Table 3, off‐line social support had a large negative elevation in the SSM analysis, reflecting its large inverse correlation with total interpersonal problems (see Table 2). The SSM model fit was excellent, and the amplitude value suggests specific interpersonal content. The angular displacement and associations with IPC dimensions indicate that that the interpersonal style associated with higher offline support is clearly warm and somewhat dominant (see Figure 2, right panel). Given the inverse association with problem severity, this theme is indicative of overall interpersonal style rather than the primary problem content.

Social anxiety had a large elevation value, consistent with its large correlation with total interpersonal problems. Model fit was adequate, and the amplitude value suggests some specific interpersonal content. The angular displacement and associations with affiliation and control IPC dimensions indicate that that the interpersonal style and major problem content associated with social anxiety is on average hostile and somewhat submissive (Figure 2, right panel).

In comparisons of online and offline measures (see Table 3), the social support scales had similar interpersonal styles (i.e., clearly warm, slightly dominant), but very different associations with overall interpersonal problems; higher offline support was strongly associated with lower levels of general interpersonal problems, whereas online support had a small positive association, elevation difference = −0.52 (CI: −0.58, −0.46). The association of social anxiety with overall interpersonal problems was similar to the association for online negativity, elevation difference = 0.02 (CI: −0.01, 0.05) and somewhat larger than the nonetheless substantial association of internet addiction with general interpersonal problems, elevation difference = 0.14 (CI: 0.10, 0.19).

Associations of offline social support and social anxiety with interpersonal problems were generally similar for men and women and across age groups, with some exceptions (see Tables S1 and S2). For example, social anxiety was strongly associated with greater reported interpersonal difficulties and a hostile‐submissive style among younger and older participants, but its strong association with general interpersonal distress was accompanied by a more heterogeneous (i.e., less specific) interpersonal style among middle‐aged participants.

## 6 Discussion

The current study examined associations of self‐reported online social experiences and excessive social media use with interpersonal problems, and compared these associations with those for offline support and social anxiety. As predicted, online social negativity and excessive social media use were strongly associated with more severe interpersonal problems, but not with a specific interpersonal style or problem area. The association of online social negativity and excessive social media use with general interpersonal problems or distress was similar in magnitude to the well‐established, large association for social anxiety (e.g., Cain et al. 2010).

Both on‐line and off‐line social support were associated with a warm and somewhat dominant interpersonal style. However, online social support was significantly, albeit weakly, associated with greater interpersonal difficulties, a clear contrast to the current and previous evidence (Osman et al. 2014) of an inverse association for offline support. This may indicate that online support comes with the risk of exposure to greater negative TMSE (Uchino et al. 2024). To the extent that SSM amplitude and displacement values reflect the content or theme of the most prominent interpersonal problems, rather than interpersonal style alone, the results suggest that online support tends to be associated with difficulties being too intrusive, self‐disclosing, and nurturing. The differing associations of online versus offline social support with interpersonal distress are similar to their associations with depression (Shensa et al. 2020).

To our knowledge, this is the first use of the interpersonal perspective in personality and clinical psychology (Wright et al. 2023)—in general and specifically via IPC‐based measures of broader interpersonal difficulties—to examine associations of TMSE and excessive social media use with overall social functioning. The results indicate that adverse TMSE and excessive social media use have strong associations with broader psychosocial functioning, and are not context‐specific difficulties. Similar findings have been reported in which rejection sensitivity in off‐line and on‐line contexts are distinguishable but substantially correlated (Andrews et al. 2022).

Follow‐up analyses revealed several potentially important demographic differences. Associations of online negativity and excessive social media use with general interpersonal problems were large for both men and women, but significantly larger for men. The association of online social support with greater interpersonal problems was significant only among men. Thus, the interpersonal perspective on online experiences is relevant for both genders, but particularly so for men. Further, the general interpersonal difficulties associated with negative TMSE are apparent for all age groups, but the associations are smaller among older individuals, and online social support was associated with *lower* general interpersonal distress in this group.

## 7 Constraints on Generality and Other Limitations

Recruitment via Qualtrics could limit generalizability of the findings. However, Qualtrics‐obtained panel data has been found to be generalizable to traditionally recruited community samples for video game addiction research (Belliveau et al. 2022). It is difficult to determine the extent of clinically meaningful levels of distress and/or dysfunction in the present sample, as some measures lack adequate normative and clinical population values. However, levels of social anxiety in the sample (see Table 1) suggest that individuals with clinically meaningful levels of symptomatology are included (c.f., Brown et al. 1997).^1^

Importantly, there were too few sex and gender minorities to permit generalization to an important segment of the population that experiences both benefits and risks from social media use (Chan 2023). Further, there is growing concern that social media use confers risks for reduced adjustment, well‐being, and physical health among children and adolescents, especially symptoms of anxiety and depression (Fassi et al. 2024; Khetawat and Steele 2023). The present results cannot be generalized to this age range but IPC‐based assessments are available for future research on related issues in children and adolescents (e.g., Trucco et al. 2013). These methods could help determine the extent to which difficulties with social media are associated with broader interpersonal processes in this vulnerable population.

Some evidence suggests that associations between social media use and adjustment observed in cross‐sectional, between‐person analyses differ from those emerging from within‐person prospective analyses (Stavrova and Denissen 2021; Tuck and Thompson 2024a), and implicit causal models in which social media use influences psychosocial adjustment may be mis‐specified by ignoring confounding factors (Sewall and Parry 2024). Hence, conclusions based on cross‐sectional analyses such as those in the current study may be misleading. Further, the associations of online experiences with interpersonal problems observed here may be inflated by common method variance. Also, different uses of social media (e.g., sharing opinions vs. social comparisons) have different emotional correlates and consequences (Tuck and Thompson 2024b; Vaid and Harari 2021), and they may be related to different interpersonal patterns, as well.

## 8 Conclusions and Future Directions

Rather than context‐specific difficulties, the present results suggest that negative TMSE and excessive social media use are strongly associated with general interpersonal problems. Thus, the online context may be “another venue” in which broader “dysfunctional interpersonal interactions” are evident (Feinstein et al. 2012, p. 356), perhaps especially for men and less so for older adults. Certainly, there are unique ways in which TMSE and related online behavior might influence emotional adjustment, health and well‐being that require conceptual models and methods specific to these contexts. Nonetheless, the present results illustrate the potential value of the interpersonal perspective (Wright et al. 2023) for efforts to explicate the nature and consequences of online behavior and experiences. In this perspective, personality, emotional adjustment, and quality of relationships are evident in recurring patterns of interpersonal situations, behavior, and experience (Wright et al. 2023). Interpersonal situations and experiences comprise a broad range of contexts and forms, including in‐person interactions and mental representations of such interactions, such as rumination about previous interactions and worry about future interactions. TMSE and related processes clearly fall within the scope of this perspective. Importantly, the current results suggest at least a moderate degree of association in these interpersonal patterns across online and more general contexts.

In the interpersonal framework, status (e.g., respect) and inclusion (e.g., belonging, acceptance) are fundamental motives (Anderson et al. 2015; Baumeister and Leary 1995), corresponding to the main IPC dimensions. Status and inclusion are also independent influences on self‐esteem (Mahadevan et al. 2019) and key commodities in social exchange (Foa and Foa 2012), potentially evident in online interactions. For example, as a prototypical expression of hostile‐dominance, cyberbullying would deny both status and acceptance to the target while implicitly self‐granting both social commodities to the perpetrator (Wiggins and Trapnell 1996).

The interpersonal perspective also describes how moment to moment interactions can be maintained over time. The complementarity principle posits that expressions of warmth invite or evoke warmth in return from interaction partners, and hostility evokes hostility (e.g., Fox et al. 2021). These recurring patterns are further explicated in the concept of transaction cycles in which an individual's internal cognitive and affective characteristics (e.g., appraisals, expectations, emotions) promote specific expressive behaviors varying along the IPC dimensions, which in turn activate cognitive and affective processes in interaction partners that promote complementary expressive behaviors (Wright et al. 2023). In this way, both healthy and dysfunctional patterns are maintained and potentially strengthened over time. Important aspects of TMSE and online behavior vary along the broad IPC dimensions, and recurring patterns of online behavior and experience may affect emotional adjustment, health and overall well‐being to the extent that they enhance or threaten the individual's perceived status and inclusion. Future research could examine these interpersonal aspects of online behavior and experience.

Future research could also explore applied implications of an interpersonal perspective on online experiences. For individuals seeking mental health services for problematic TMSE or excessive social media use, formal assessments may include measures directly related to the online context (e.g., Andrews et al. 2022; Andreassen et al. 2016; Hall et al. 2021; Kent de Grey et al. 2019). Assessment research could examine the extent to which IPC‐based measures of broader interpersonal functioning have incremental utility in case conceptualization and intervention. For intervention research, a variety of treatments are useful in reducing problematic social media use and related distress, but further development of theory‐driven approaches is important (Herriman et al. 2024). Simple restriction‐based interventions are less effective than more involved counseling, mindfulness or CBT approaches (Plackett et al. 2023). In CBT and other interventions for common psychological concerns, the overall severity of interpersonal problems is reduced, even without an explicit interpersonal focus in treatment (McFarquhar et al. 2018). However, more pronounced interpersonal problems at the outset of therapy, indexed as the IIP total score analogous to elevation in the SSM, predict less improvement (Gómez Penedo and Flückiger 2023). Future research could evaluate the extent to which interventions for problematic TMSE improve general interpersonal functioning, and whether augmentation with interpersonally‐focused intervention components enhances outcomes.

Potential implications of the interpersonal perspective for assessment and intervention must be supported by additional research before any serious consideration in clinical services, as the current results are quite preliminary and limited as discussed above. In the meantime, for social media research more broadly, the concepts and methods of the interpersonal perspective may be useful in efforts to understand the role of the online context in emotional adjustment, social relations, and overall well‐being.

## Ethics Statement

This research was approved by the Institutional Review Board of Indiana State University (#1689180), and all participants provided informed consent via the online survey procedure.

## Conflicts of Interest

The authors declare no conflicts of interest.

## Supporting information

SupplementFigure1.

SupplementFigure2.

SupplementTable1.

SupplementTable2.

SupplementTextOnly.

## Endnotes

1 In post‐hoc analyses (see Figures S1 and S2), linear and curvilinear associations with interpersonal problem severity were significant for negative online experiences (linear *β* = 0.67, *t*(1335) = 25.56, *p *< 0.001; curvilinear β = 0.07, *t*(1335) = 2.86, *p* = 0.004) and excessive social media use (i.e., social media addiction) (linear *β* = 0.52, *t*(1334) = 22.13, *p* < 0.001; curvilinear *β* = 0.14, *t*(1334) = 5.81, *p*< 0.001), indicating that the magnitude of these large positive associations increased at higher levels of negative TMSE and excessive social media use, perhaps supporting their clinical relevance. That is, associations with interpersonal problems were stronger at higher levels of online difficulties, although these curvilinear effects were small, especially for negative online experiences. The curvilinear effects were clearly evident for both men and women, but for social media addiction this effect was particularly apparent for men (Figure S2), again suggesting the interpersonal perspective is relevant for both genders but especially for men.
