# Supplementary figures and images for: Are Online Social Experiences Associated With General Interpersonal Problems? A Circumplex Assessment
Source: J Clin Psychol. 2026 Apr 3;82(8):1166–76. doi: 10.1002/jclp.70142 (PMC13341040; doi:10.1002/jclp.70142)

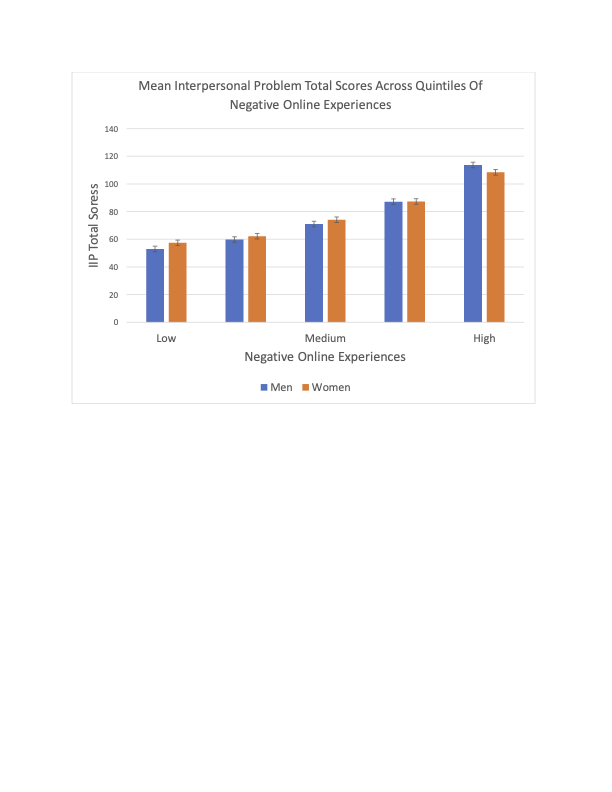

Supplement: Supplementary file 1 — SupplementFigure1. [file JCLP-82-1166-s005.tiff]

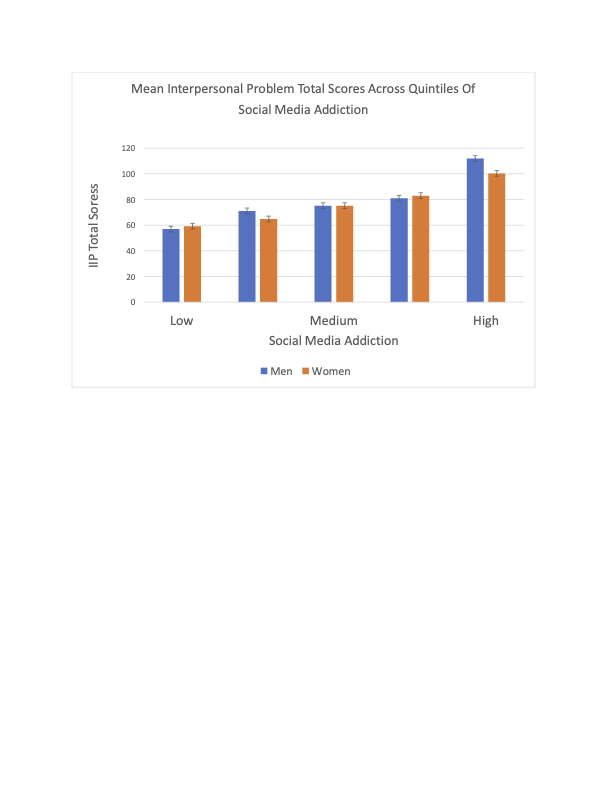

Supplement: Supplementary file 2 — SupplementFigure2. [file JCLP-82-1166-s003.tiff]
