# Supplementary material for: Are Online Social Experiences Associated With General Interpersonal Problems? A Circumplex Assessment
Source: J Clin Psychol. 2026 Apr 3;82(8):1166–76. doi: 10.1002/jclp.70142 (PMC13341040; doi:10.1002/jclp.70142)
Supplement: Supplementary file 3 — SupplementTable1. [file JCLP-82-1166-s004.docx]

**Men**

| **Scale** | **Fit** | **Elevation** | **Amplitude** | **Displacement** | **Affiliation** | **Control** |
| --- | --- | --- | --- | --- | --- | --- |
| Online Support | .97 | .21(.14, .29) | .11(.08, .15) | 17.2(8.0, 25.8) | .13(.10, .16) | .04(.02, .06) |
| Online Negativity | .33 | .68(.63, .72) | .03(.02, .05) | 115.6(79.1, 153.5) | -.01(-.03, .01) | .03(.01, .03) |
| Internet Addiction | .93 | .53(.47, .59) | .05(.03, .07) | 37.3(15.6, 62.2) | .04(.01, .06) | .03(.01, .05) |
| Offline Support | .86 | -.44(-.49, -.39) | .13(.10, .16) | 11.9(.06, 22.2) | .13(.10, .16) | .03(.00, .05) |
| Social Anxiety | .63 | .68(.63, .73) | .07(.06, .10) | 196.3(184.3, 208.5) | -.07(-.09, -.05) | -.02(-.04, -.01) |

**Women**

| **Scale** | **Fit** | **Elevation** | **Amplitude** | **Displacement** | **Affiliation** | **Control** |
| --- | --- | --- | --- | --- | --- | --- |
| Online Support | .97 | -.01(-.07, .06) | .11(.08, .15) | 25.1(7.9, 42.3) | .10(.06, .13) | .05(.01, .08) |
| Online Negativity | .52 | .52(.47, .57) | .09(.06, .11) | 105.3(88.2, 122.4) | -.02(-.05, .00) | .08(.06, .11) |
| Internet Addiction | .43 | .42(.36, .47) | .06(.04, .09) | 72.3(47.5, 100.7) | .02(-.01, .05) | .06(.04, .09) |
| Offline Support | .93 | -.38(-.42, -.33) | .15(.11, .18) | 20.8(8.7, 33.3) | .14(.10, .17) | .05(.02, .08) |
| Social Anxiety | .78 | .57(.52, .62) | .10(.08, .13) | 211.7(195.9, 229.4) | -.13(-.18, -.08) | -.08(-.13, -.04) |
