# Supplementary material for: Are Online Social Experiences Associated With General Interpersonal Problems? A Circumplex Assessment
Source: J Clin Psychol. 2026 Apr 3;82(8):1166–76. doi: 10.1002/jclp.70142 (PMC13341040; doi:10.1002/jclp.70142)
Supplement: Supplementary file 4 — SupplementTable2. [file JCLP-82-1166-s002.docx]

**Younger Participants**

| **Scale** | **Fit** | **Elevation** | **Amplitude** | **Displacement** | **Affiliation** | **Control** |
| --- | --- | --- | --- | --- | --- | --- |
| Online Support | .95 | .03(-.07, .13) | .12(.08, .17) | 7.1(350.6, 21.9) | .12(.08, .17) | .02(-.02, .05) |
| Online Negativity | .33 | .56(.49, .62) | .04(.01, .07) | 155.5(103.5, 203.1) | -.03(-.07, -.00) | .02(-.01, .04) |
| Internet Addiction | .69 | .32(.22, .42) | .03(.01, .07) | 22.4(288.1, 139.1) | .03(-.01, .06) | .01(-.02, .06) |
| Offline Support | .88 | -.41(-.46, -.35) | .16(.12, .21) | 24.2(11.6, 37.8) | .15(.11, .19) | .07(.03, .10) |
| Social Anxiety | .87 | .57(.50, .63) | .15(.12, .19) | 210.5(190.2, 209.5) | -.13(-.17, -.10) | -.08(-.11, -.05) |

**Middle-Aged Participants**

| **Scale** | **Fit** | **Elevation** | **Amplitude** | **Displacement** | **Affiliation** | **Control** |
| --- | --- | --- | --- | --- | --- | --- |
| Online Support | .96 | .24(.16, .32) | .11(.08, .15) | 26.3(10.9, 41.3) | .10(.07, .13) | .05(.02, .08) |
| Online Negativity | .41 | .67(.61, .72) | .05(.03, .07) | 113.5(84.1, 141.5) | -.02(-.04, .00) | .05(.02, .07) |
| Internet Addiction | .69 | .56(.48, .62) | .05(.02, .08) | 52.3(26.1, 86.0) | .03(.00, .06) | .04(.01, .06) |
| Offline Support | .83 | -.45(-.51, -.40) | .11(.08, .14) | 18.2(2.0, 35.0) | .10(.07, .14) | .03(.00, .07) |
| Social Anxiety | .54 | .67(.62, .72) | .06(.04, .09) | 186.8(169.8, 202.9) | -.06(-.08, -.04) | -.01(-.03, .01) |

**Older Participants**

| **Scale** | **Fit** | **Elevation** | **Amplitude** | **Displacement** | **Affiliation** | **Control** |
| --- | --- | --- | --- | --- | --- | --- |
| Online Support | .94 | - .10(-.17,-.02) | .15(.10, .19) | 27.7(10.1, 45.2) | .13(.08, .18) | .07(.02, .11) |
| Online Negativity | .67 | .35(.25, .45) | .10(.07, .14) | 99.0(75.6, 126.3) | -.02(-.06, .03) | .10(.06, .13) |
| Internet Addiction | .41 | .29(.20, .37) | .06(.03, .10) | 64.7(26.5, 109.8) | .03(-.02, .07) | .05(.02, .09) |
| Offline Support | .95 | -.30(-.36, -.23) | .18(.13, .23) | 10.2(355.4, 24.6) | .18(.13, .23) | .03(-.01, .08) |
| Social Anxiety | .92 | .48(.40, .55) | .15(.11, .21) | 219.3(203.5, 234.7) | -.12(-.16, -.08) | -.10(-.15, -.05) |
