# Supplementary material for: Are Online Social Experiences Associated With General Interpersonal Problems? A Circumplex Assessment
Source: J Clin Psychol. 2026 Apr 3;82(8):1166–76. doi: 10.1002/jclp.70142 (PMC13341040; doi:10.1002/jclp.70142)
Supplement: Supplementary file 5 — SupplementTextOnly. [file JCLP-82-1166-s001.docx]

**Supplemental Tables.** Structural Summary Statistics with 95% CIs for associations of online social support, online social negativity, internet addiction, offline social support and social anxiety scales with self-reported interpersonal problems for men and women separately (Supplemental Table 1), and for Younger, Middle-aged and Older Participants, separately Supplemental Table 2).

**Supplemental Table 1**

**Supplemental Table 2**

Supplemental Depiction of Curvilinear Associations of Online Negative Experiences (Figure S1) and Excessive Social Media Use (i.e., Social Media Addiction) (Figure S2) with Total Interpersonal Problem Severity

In Footnote 1 in the main text, continuous linear and curvilinear effects were tested via multiple regression. To illustrate the effect here, quintiles on the predictor variable are created. The primary analyses did not reveal a Gender x Negative Online Experiences interaction. However, given that the Gender x Excessive Social Media Use (i.e., Social Media Addiction) interaction was significant in the in the primary analyses (see main text), the main effect of Excessive Social Media Use and separate comparisons within genders are presented and discussed for Figure S2.

Figure S1

Note: Differences in IIP Total scores are significantly different between each of the first four quintiles of Negative Online Experiences (all p<.001), with the magnitude of these differences becoming progressively larger (Cohen’s d = .25, .52, .65). However, the difference between the 4^th^ and 5^th^ groups is notably larger (Cohen’s d = 1.02). That is, the association between Negative Online Experiences and overall interpersonal difficulty is considerably stronger at the higher levels of online negativity.

Figure S2

Note: For both men and women, differences in IIP Total scores are significantly different between each of the first four quintiles of Social Media Addiction (i.e., excessive social media use (all p<.001), Cohen’s d = .54, .15, .22 for men, and .21, .39, .29 for women. However, the difference between the 4^th^ and 5^th^ groups is notably larger, especially for men, Cohen’s d = 1.17 for men, .65 for women. That is, the association between Social Media Addiction and overall interpersonal difficulty is stronger at the higher levels of excessive social media use for both genders, but this curvilinear association is particularly evident among men.
